# Randomised Controlled Trial of a Customised Text Messaging and Activity Monitor Program for Lifestyle Improvement after Gestational Diabetes

**DOI:** 10.3390/nu16060820

**Published:** 2024-03-13

**Authors:** Ngai Wah Cheung, David Simmons, Simone Marschner, Aravinda Thiagalingam, Dharmintra Pasupathy, Ben J. Smith, Victoria Flood, Mark McLean, Sarah J. Melov, Roslyn Hogan, Suja Padmanabhan, Anna Duke, Cellina Ching, Haeri Min, Justin McNab, Clara K. Chow

**Affiliations:** 1 Westmead Applied Research Centre, Faculty of Medicine and Health, University of Sydney, Sydney, NSW 2006, Australia; simone.marschner@sydney.edu.au (S.M.); aravinda.thiagalingam@sydney.edu.au (A.T.); ben.smith@sydney.edu.au (B.J.S.); vicki.flood@sydney.edu.au (V.F.); cellina.ching@health.nsw.gov.au (C.C.); haeri.min@sydney.edu.au (H.M.); clara.chow@sydney.edu.au (C.K.C.); 2 Department of Diabetes and Endocrinology, Westmead Hospital, Sydney, NSW 2145, Australia; roslyn.hogan@health.nsw.gov.au (R.H.); suja.padmanabhan@sydney.edu.au (S.P.); 3 Reproduction and Perinatal Centre, Faculty of Medicine and Health, University of Sydney, Sydney, NSW 2006, Australia; dharmintra.pasupathy@sydney.edu.au (D.P.); sarah.melov@health.nsw.gov.au (S.J.M.); justin.mcnab@sydney.edu.au (J.M.); 4 Macarthur Diabetes Service, Campbelltown Hospital, Campbelltown, NSW 2560, Australia; da.simmons@westernsydney.edu.au; 5 School of Medicine, Western Sydney University, Campbelltown, NSW 2560, Australia; 6 Department of Cardiology, Westmead Hospital, Sydney, NSW 2145, Australia; 7 Westmead Institute for Maternal & Fetal Medicine, Women’s & Newborn Health, Westmead Hospital, Sydney, NSW 2145, Australia; 8 Sydney School of Public Health, Faculty of Medicine and Health, The University of Sydney, Sydney, NSW 2006, Australia; 9 The University Centre for Rural Health, Northern Rivers, Faculty of Medicine and Health, The University of Sydney, Lismore, NSW 2480, Australia; 10 Department of Diabetes and Endocrinology, Blacktown Hospital, Blacktown, NSW 2148, Australia; mark.mclean@health.nsw.gov.au (M.M.); anna.duke@health.nsw.gov.au (A.D.)

**Keywords:** gestational diabetes, diabetes prevention, lifestyle program, M-health, text-messaging, activity monitor, randomised controlled trial

## Abstract

Gestational diabetes (GDM) is associated with a long-term risk of diabetes. We aimed to determine whether a text-messaging-based lifestyle support program would improve diabetes risk factors following GDM. Women with GDM were randomised following delivery to receive four text messages per week supporting a healthy lifestyle and parenting for 6 months, with feedback from an activity monitor (intervention), or to receive the activity monitor only (control). The primary outcome was a composite of weight, physical activity and dietary goals. There were 177 women randomised, with 88 intervention and 89 control participants. All the participants experienced COVID-19 lockdowns during the study. Six-month primary outcome data were obtained for 57 intervention participants and 56 controls. There were 7/57 (12%) intervention and 6/56 (11%) control participants who met the primary outcome (relative risk, 1.08; 95%CI, 0.63–1.85; *p* = 0.79). Two intervention participants met the dietary goals compared to none of the control participants (*p* = NS). The intervention participants were more likely to record >1000 steps/day (on 102 ± 59 vs. 81 ± 59 days, *p* = 0.03). When analysed monthly, this was not initially different but became significant 3–6 months post-partum. Interviews and surveys indicated that with the Intervention, healthier choices were made, but these were negatively impacted by COVID-19 restrictions. Participants found the messages motivational (74%) and the activity monitor useful (71%). In conclusion, no improvement in the diabetes risk factors occurred among the women receiving the text messaging intervention when affected by COVID-19 restrictions.

## 1. Introduction

Gestational diabetes (GDM) is the most common medical complication in pregnancy, affecting up to a quarter of all pregnancies under the World Health Organization (WHO) criteria, and is associated with poorer maternal and child-related outcomes [1]. Whilst glucose tolerance usually normalises following delivery, women with GDM have a high risk of recurrent GDM and future type 2 diabetes. About half will develop diabetes eventually, depending on concurrent risk factors and the diagnostic criteria used [2]. Meta-analyses show that women with GDM are 6–10 times more likely to develop diabetes compared to women without a history of GDM [3,4]. Population estimates suggest women with GDM may contribute to about a third of incident type 2 diabetes [4]. Therefore, targeting women with GDM is imperative to addressing the burden of diabetes.

Women with past GDM in the Diabetes Prevention Program (DPP)—a randomised controlled trial (RCT) of intensive lifestyle intervention for people later in life with impaired glucose tolerance—achieved a 53% risk reduction in diabetes [5]. The RCTs to date on lifestyle interventions in the post-partum period have generally been small and under-powered and have not shown reductions in diabetes incidence. However, a 2020 meta-analysis of 10 studies found lifestyle interventions implemented within 3 years after GDM pregnancy reduced the risk of post-partum diabetes (relative risk 0.57, 95% confidence interval 0.42–0.78) [6]. Of note, this meta-analysis did not include a recent large RCT on a 12-month pragmatic lifestyle intervention for preventing glycaemic deterioration among 1612 South Asian women with recent GDM, which failed to demonstrate a benefit [7]. The Gestational Diabetes’ Effect on Moms (GEM) study cluster-randomised 2280 women with GDM to receive a DPP style intensive intervention or the usual care over 6 months but was not powered to detect a change in the incidence of diabetes [8]. However intervention participants were more likely to meet weight goals or have greater increases in physical activity (PA) at 6 months.

While these studies suggest that lifestyle modification programs for the prevention of diabetes following a GDM pregnancy are beneficial, these interventions are often resource-intensive and expensive. Therefore, they have not been translated into routine care. Affordable, scalable and accessible interventions are needed. Our goal is to develop a sustainable intervention readily translatable into clinical practice to reduce diabetes risk amongst women who have had GDM using simple everyday technologies. We recently conducted a feasibility study of 60 women with GDM, randomised to receive a 6-month post-partum intervention comprising healthy lifestyle text messaging and an activity monitor or the usual care [9]. The content was delivered at a low cost via text messages and integrated with an activity monitor. It was highly acceptable to most of the women, with the majority giving positive feedback. Text-messaging-based programs have also been used in interventions to improve lifestyle and cardiovascular risk factors [10,11,12,13] and, among people with T2DM, have been shown to reduce body mass index (BMI) and HbA1c [14,15]. Further, text messaging programs have been shown to be cost-effective [16].

The aim of the current study is to build on the pilot study and determine whether a text messaging support program, integrated with feedback from activity monitors, will improve the diabetes risk factors of PA, healthy diet and weight management following a GDM pregnancy.

## 2. Materials and Methods

Smart Mums with Smart Phones 2 (SMs2) compared women randomised to either receive an activity monitor and customised education and support via text messaging (intervention) or usual care with an activity monitor only (active control) for 6 months after a GDM pregnancy. SMs2 was registered on the Australian New Zealand Clinical Trials Registry ACTRN12620000615987. The study was conducted according to the guidelines of the Declaration of Helsinki and approved by the Western Sydney Local Health District Human Research Ethics Committee (2019/ETH13240, 23 July 2020). Informed consent was obtained from all the subjects involved in the study.

**Figure 1:**
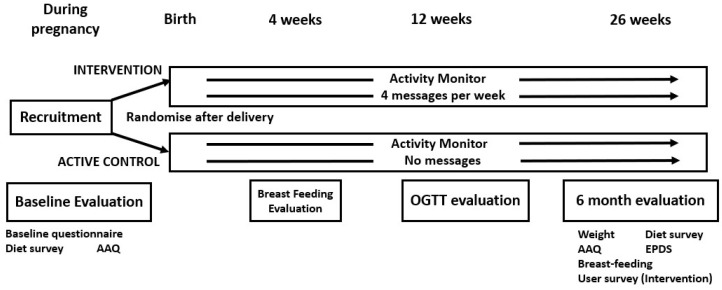
Trial design, including evaluations. AAQ = Active Australia Questionnaire, OGTT = oral glucose tolerance test, EPDS = Edinburgh Postnatal Depression Scale.

The trial design has previously been described [17] but is summarised in Figure 1 and below.

### 2.1. Participants

Three tertiary care hospitals in Western and South Western Sydney participated in the study. Together, these hospitals manage about 3000 cases of GDM per year. The hospitals all have dedicated diabetes in pregnancy services, and women with GDM received multidisciplinary care from endocrinologists, diabetes educators, dietitians, obstetricians and midwives. At all 3 hospitals, women received training in self-management from diabetes educators and dietitians, and endocrinologists would manage the women with insulin when needed, or Metformin. Participants with GDM were recruited whilst pregnant and during routine antenatal care by research assistants. Women diagnosed with GDM based on either the WHO [18] criteria (1 hospital) or the 1998 Australasian Diabetes in Pregnancy Society [19] criteria (2 hospitals) were accepted.

Women were eligible if they owned a smart phone with internet access, were aged > 18 years old and were able to read text messages in English. Women were excluded if they were already using an activity monitor, had pre-existing diabetes or an oral glucose tolerance test (OGTT) result in the “diabetes mellitus in pregnancy” range (fasting glucose ≥ 7.0 mmol/L and/or 2 h glucose ≥ 11.1 mmol/L) in the first 20 weeks of pregnancy, planned to spend >1 month overseas, were on medications affecting glucose metabolism (other than treatment for GDM), had a twin pregnancy, had a baby with a fetal disorder likely to require increased care or were physically unable to walk regularly.

### 2.2. Randomisation

Randomisation occurred immediately after delivery. This enabled discontinuation of the participant prior to randomisation in the event of a major complication whereupon the receipt of text messages would be inappropriate.

Computerised randomisation into the intervention or control group (1:1) was stratified by site, using permuted blocks of sizes 4 and 6. Randomisation codes were generated using the randomizeR package in R (ver. 3.5.2).

### 2.3. The Activity Monitor

Both the intervention and control participants received a wrist-worn activity monitor (Garmin Vivofit 4^®^, Garmin Ltd., Olathe, KS, USA) and installed the manufacturer’s app onto their smart phones. Data from the activity monitors were uploaded from the app to the Garmin servers. This was downloaded daily by the study team for customisation of the messages and analysis.

### 2.4. Intervention

The intervention comprised a patient-centred lifestyle program using semi-personalised (addressing the participant by name in the messages) and customised (see below) mobile phone text messages, facilitated by the activity monitor data. The message management engine selected messages from message banks based on prespecified algorithms. The intervention was designed giving consideration to theoretically based mechanisms of action that have been used to achieve lifestyle changes in chronic disease prevention, including the Theory of Reasoned Action, Social Cognitive Theory and the Health Belief Model [20].

The intervention participants received 4 text messages per week beginning 1–2 weeks post-partum. Whilst the intent was that the messaging would be unidirectional, reply messages were monitored, and those of a clinical nature were escalated to a physician for review and action if necessary.

### 2.5. Content of the Messages

The text messages related to (i) PA; (ii) healthy eating; (iii) parenting, breastfeeding and infant health; and (iv) feedback based on the activity monitor. The initial messages focused more on issues relevant to early parenting, but over time, this shifted towards a long-term maternal healthy lifestyle. The early lifestyle messages promoted the adoption of healthy lifestyle behaviours, whilst the later messages concentrated on supporting the maintenance of changes. The PA messages were designed to gradually motivate the women to achieve at least 5 days of >10,000 steps/day each week and 30 min of moderate-intensity activity on most days. The dietary messages supported the Australian Dietary Guidelines [21] and healthy eating to reduce weight and diabetes risk. Parenting and infant health messages addressed issues such as breastfeeding, weaning, infant care, sleep, allergies and mental health. The messages were timed to align with appropriate infant age-specific content, such as reminders for vaccination. Some messages included links to public websites belonging to reputable health or government organisations where the participant could obtain more detailed information regarding the subject of the message. Emojis were included within the text messages, as they have been shown to help bridge cultural diversity and modernise digital interventions [22]. Three early messages reminded participants to undertake an OGTT at 6–12 weeks post-partum, as per the Australian guidelines [23].

Examples of the messages include, “Hi <XXX>, getting back into exercise? Start small and gradually increase time/pace/distance as you become more fit. Keep up the good work!” (personalised physical activity message); “Include plenty of veggies in your main meal, at least half the plate” (healthy eating message); “Latching baby maybe difficult-for help try https://globalhealthmedia.org/portfolio-items/attaching-your-baby-at-the-breast/, accessed on 11 March 2024 or contact your child and family health nurse” (parenting message with a web link); “Fantastic stepping—over target 🌻! We are increasing your target to <XXX> steps a day” (activity-monitor-related message with an emoji).

Because of the COVID-19 lockdowns mandated from June 2021, 17 messages were changed mid-trial. Messages which encouraged group activity were replaced, and new messages indicated that it was acceptable to delay the 6–12 week OGTT under these circumstances.

### 2.6. Message Customisation

The messages were customised to breastfeeding status and adjusted based on periodic surveys. PA coaching was individually customised utilising continuously updated participant activity monitor data. The participants received a weekly text message with adaptive step targets, encouragement and reminders based on their activity monitor data. For the first 10 weeks post-partum, the daily step target was set at 3500. Using a rank order percentile algorithm, an incremental daily step target was set each week based on the number of steps taken in the previous 2 weeks [9,24,25]. The maximum target was 10,000 steps a day.

### 2.7. Active Control

The control participants received a welcome text message and administrative messages such as requests to complete surveys for the evaluation but not the intervention messages.

### 2.8. Data Collection

Data were collected at baseline and at 4, 12 and 26 weeks post-delivery. The baseline data were collected by research assistants at recruitment, which was prior to delivery and randomisation. The birth and pregnancy data were collected immediately after delivery by the research assistants but with blinding maintained for participant randomisation. The post-partum data, including most of the outcomes, were self-reported through online surveys via a text message link. Text reminders were sent to participants who did not complete their surveys in a timely manner, and at least 3 attempts to contact them by telephone were made if the 6-month surveys were still not completed. The OGTT results were verified with pathology providers.

A short food frequency questionnaire was used to assess participant dietary intake of fruits, vegetables and discretionary foods. Discretionary foods included soft drinks, takeaway, cakes, ice cream, hot chips, confectionary and alcohol, all of which were specifically asked about in the diet survey. The Active Australia Questionnaire (AAQ) [26] was used to calculate the duration of PA, using the formula [walk time + moderate physical activity time + 2 × (vigorous activity time)]. The baseline dietary survey and AAQ asked about diet and PA prior to pregnancy. The BLIiNG survey applying the Breastfeeding Length and Intensity Scoring System (BLISS) was used to assess breastfeeding [27]. The likelihood of postnatal depression was assessed using the Edinburgh Postnatal Depression Scale [28]. A score of ≥10 in the EPDS was taken to indicate the possibility of postnatal depression.

### 2.9. The Study Outcomes

The primary outcome of the study was a composite of weight, dietary and PA outcomes, evaluated at 6 months. A composite outcome was chosen, as the size and duration of the study would not yield a significant difference in diabetes status or in any individual component of the composite outcome. This “Healthy Lifestyle Outcome” (HLO) was considered to have been met if at least two of the following three components were achieved: 1. Weight: Reaching pregravid weight if the pregravid BMI was <25 kg/m^2^ or losing 5% of pregravid weight if the pregravid BMI was ≥25 kg/m^2^, as described in the GEM trial [8]; 2. PA: Whether the Australian guidelines of 150 min of moderate-intensity PA each week had been met, as determined using the AAQ [25]; 3. Diet: Whether 1 serving of fruit and 3 servings of vegetables were consumed per day and discretionary foods were consumed ≤14 times a week. Our diet criteria were less ambitious than the health guidelines [21], as we have found that this population is unlikely to reach the guideline dietary targets at this time [9,29,30].

The secondary outcomes included the three individual components of the HLO, BMI, weekly minutes of moderate-intensity PA, the duration and intensity of breastfeeding, the likelihood of postnatal depression, the use of the activity monitor and whether an OGTT had been performed by 12 weeks post-partum. In addition, the components of the dietary outcome were analysed. The number of steps taken as measured using the activity monitor was not included as an outcome, as people in the control arm who wear the activity monitor are more likely to be active than those who do not wear it, resulting in bias in the step count. However, we examined the usage of the activity monitor between the two arms of the trial. As it is not possible to determine whether a lack of steps on a day was due to sedentariness or a failure to wear the activity monitor, we set a daily step count of >1000 steps as a marker of substantial usage of the activity monitor on a given day.

Information was collected about the acceptability and use of the text messages among the intervention participants, and the use of the activity monitor by all participants.

### 2.10. Qualitative Interviews

Focus groups were planned, but due to COVID restrictions, individual semi-structured qualitative telephone interviews with the intervention participants were conducted instead. These explored the barriers and enablers to program participation and involved receiving feedback about the intervention components. Participants were selected and invited sequentially from those who had most recently completed the study.

The interviews commenced with the initial question “Can you tell me about your experience on the SMs2 program?”. The interviews ceased upon thematic saturation when sufficient information was obtained on diverse opinions. A single author (S.J.M.), who had no previous relationship with any of the participants, conducted all the interviews. All of the interviews were audio-recorded with consent. A professional transcription service was utilised. A thematic approach was used for the analysis with six non-sequential phases. Familiarisation with the data, coding and theme development was conducted by two of the authors (S.J.M. and J.M.). The collection, analysis and presentation of in-depth, detailed and contextualised data, paying critical attention to matters of reflexivity in the interview process, enhanced rigour/validity, trustworthiness and quality. The quality of the data coding analysis and considerations to reduce bias was further strengthened by the author J.M. evaluating, cross-checking and, where necessary, reassessing both the coding frames and coded text in the iterative dialogue with the author S.J.M.

Analysis and data management was assisted using NVivo (release 1.5.1).

### 2.11. Statistical Analysis

Based on our earlier study [9], we estimated that 21% of the control participants would meet the HLO at 6 months. With 180 participants, we would have 80% power to detect an increase of 20% in the proportion meeting the primary outcome, at a significance level of 0.05, with 10% dropout.

The participants were deemed to have met the HLO if two components had achieved the target, even if data were missing for the third component. Similarly, they were deemed not to have met the outcome if two components were negative, even if the data were missing for the third component.

We followed a pre-specified statistical analysis plan and intention-to-treat principles. The statistician team was unaware of the trial group assignments. Baseline continuous variables were presented as means and standard deviation (SD). The statistical tests were two-tailed, with a 5% significance threshold, and the outcomes were reported as means or relative risks with 95% confidence intervals. Outcome comparisons between the treatment groups were undertaken using regression models adjusting for the baseline values of the outcome measures. For dichotomous outcomes, log-binomial regression was used, and for continuous outcomes, linear regression was used. Interactions between the treatment group and baseline covariates on the primary outcome were tested by fitting a log-binomial regression, adjusting for the treatment group, covariates and an interaction term of the covariate and treatment group. The analyses were conducted using R (ver. 3.5.2, R Core Team) packages.

### 2.12. Analysis of the Activity Monitor Data

Most of the participants did not continuously wear their activity monitor, so there were gaps in the PA data collected. As it was not possible to determine whether a lack of activity was related to inactivity or that the participant had not worn the activity monitor, the following assumptions were applied to the analysis: (1) days when ≥1000 steps were recorded were indicative of the participant wearing the activity monitor for most of the day; (2) days when <1000 steps were recorded were indicative that the activity monitor had not been worn or had been minimally worn.

## 3. Results

**Figure 2:**
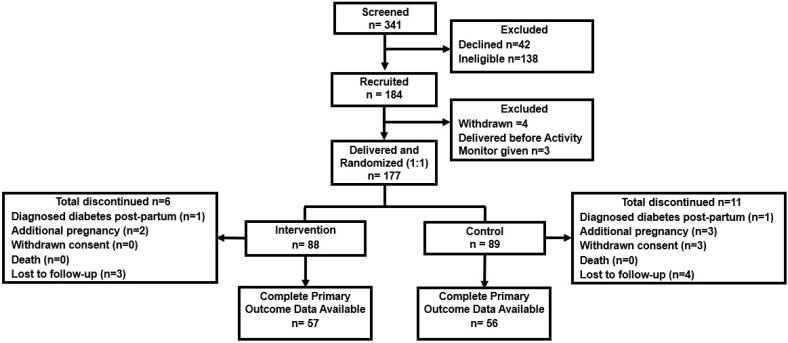
Consort diagram for the Smart Mums with Smart Phones 2 study.

There were 341 women with GDM who were screened for the study, of whom 184 were recruited and 177 randomised to the intervention and control arms of Smart Mums 2 (Figure 2). Recruitment was ceased prior to reaching the planned randomisation of 180 participants (n = 177, 88 intervention, 89 control) when part of Sydney went into complete COVID-19 lockdown and there was mandatory cessation of all face-to-face research activities in July 2021. The first participant was recruited in December 2020 with randomisation in January 2021, and the last recruitment was in July 2021 with randomisation in August 2021. Participant allocation was well matched to the baseline and pregnancy characteristics (Table 1).

### 3.1. COVID-19 Restrictions during the Study

There were varying degrees of COVID-19 restrictions during the study. Throughout most of the study, there were restrictions and quarantines on international and interstate travel and limitations placed on gatherings and community events. The catchment area of the participating hospitals went into complete COVID-19 lockdown gradually over June-July 2021, with stay-at-home orders and travel only permitted for essential activities. Outdoor exercise was limited to one hour a day. The lockdown was lifted in October 2021, though some restrictions remained in place. All the participants were affected by the major lockdown for some duration of their involvement in the study.

### 3.2. Primary and Secondary Outcomes (Intention-to-Treat Analysis)

There were 31 participants in the intervention arm and 33 in the control arm for whom complete 6-month outcome data could not be obtained. This left 57 participants in the intervention arm and 56 in the control arm with 6-month primary outcome data. The participants with primary outcome data were older, lighter, less likely to identify as being of Australian or New Zealand ethnicity, more likely to have had tertiary-level education and less likely to have been a smoker (Supplementary Material Table S1). Otherwise, there were no differences in the other baseline characteristics or pregnancy outcomes between the participants with and without primary outcome data (Supplementary Material Table S1).

There were 7/57 (12%) participants in the intervention arm and 6/56 (11%) in the control arm who met the primary outcome by achieving the HLO target at 6 months (relative risk 1.15, 95%CI 0.41–3.20), (*p* = 0.79). No interactions were identified between the treatment group and age, education, smoking, BMI and ethnicity.

No differences were detected between the intervention and control participants for any of the secondary outcomes at 6 months (Table 2). There were also no differences in any dietary outcomes between the groups (Table 3). Vegetable intake was particularly low, with only 1.2 ± 0.8 servings of vegetables consumed per day amongst the intervention participants compared to 1.0 ± 0.6 servings for the control participants (*p* = 0.29), a result which was similar to the baseline. Only four participants in the intervention group met the vegetable goal, compared to none in the control group.

There was no significant difference in the proportion of women having their post-partum OGTT within 12 weeks of delivery (intervention: 38/88 (43%) vs. control: 29/89 (33%); relative risk: 1.22; 95%CI: 0.78–1.91; *p* = 0.36).

### 3.3. Activity Monitor Usage

There were three control participants and one intervention participant who recorded no step data. Of those who used the activity monitor at all, the control participants recorded some steps on 81 ± 60 days and the intervention participants on 103 ± 64 days (*p* = 0.04). Similarly, there were more days on which the intervention participants recorded at least 1000 steps, 102 ± 59 days, compared to the control participants with 80 ± 59 days (*p* = 0.03).

**Figure 3:**
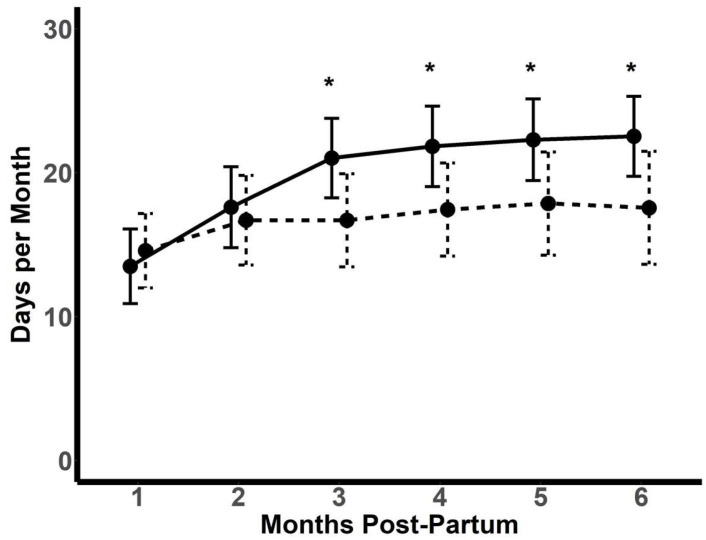
Number of days for each month post-partum that participants recorded at least 1000 steps per day. Solid line: intervention (n = 71); dashed line: control (n = 69). Data depicted as mean ± SD. * *p* < 0.05.

The number of days on which at least 1000 steps were recorded was similar for the two groups in the first two months post-partum (Figure 3). From 3–6 months, there were more days with at least 1000 steps recorded amongst the intervention participants. However, there was no difference in the mean daily steps taken between the control (4651 ± 1931) and intervention participants (4913 ± 2093) on the days where ≥1000 steps were taken (*p* = 0.46).

### 3.4. Feedback Regarding the Text Messages and Activity Monitor

Of the intervention participants, 50 provided feedback on the text messages. There were 49 (98%) who agreed that the messages were easy to understand, 37 (74%) that said the messages motivated them to improve their lifestyle and 44 (88%) that said the number of messages received each week was just right. Overall, 43 (86%) of the participants indicated that they had read at least 75% of the messages.

Both the intervention and control participants had the opportunity to provide feedback on the activity monitor. There were 73/103 (71%) who agreed that the activity monitor was useful, 61/104 (59%) who wore it most of the time and 63/104 (61%) agreed that it motivated them to change their lifestyle.

### 3.5. Qualitative Evaluation

A total of 22 intervention participants were invited to take part in the qualitative interviews at the conclusion of the program, and 14 agreed to do so.

The main theme concerning enablers was that SMs2 motivated women to make healthier choices. All the participants identified some aspect of SMs2 that was motivational for healthy choices and beneficial to their well-being post-partum. An example of such a comment is:

“…[text messages] *constantly reminding to reach our target otherwise we’re getting back the diabetes after pregnancy. Because during pregnancy it was so hard. We had to do injection on our tummy. Once I was getting the [text message] reminder I was thinking back during my pregnancy, and I was like, ‘I have to be healthy not to get diabetes, back again’*”

(Qualitative participant 10).

Further quotes from the intervention participants are given in Supplementary Material Table S2.

The main themes concerning barriers to achieving the health goals were “putting themselves last”, “lack of time” and “lack of tangible social support”. There was an absence of social support in assisting the participants with child-minding and household tasks, so there was little quarantined time for them to achieve their health goals. An example of such a comment is: “*except for my husband, there was no one [for support]*”. Eleven of fourteen (79%) participants identified that the COVID-19 lockdown periods negatively impacted their healthy eating and capacity to exercise. This was exacerbated by the home-schooling of other children and lack of childcare facilities. Involvement in paid work and study were barriers; however, these barriers would have been lessened if they had had adequate practical support. Six participants revealed that pain, generally related to caesarean birth or perineal tears, was a barrier to meeting their PA goals.

First-time mothers often identified all three supportive text message areas (diet, exercise, parenting) as beneficial. All the participants stated they would recommend the program to a friend, and most would have liked a longer program. Other suggestions for improvement included having a participant support group or network, more positive feedback, the provision of specific weight goals, having midwife-led telephone contact and educational podcasts.

### 3.6. Safety and Adverse Events

There were three cases of participant hospitalisation following randomisation, one in the control and two in the intervention arm. None were attributed to the trial interventions. There were no deaths.

## 4. Discussion

Healthy lifestyle programs developed in intervention studies for women who have had GDM have generally been resource-intensive and relatively inaccessible to women with newborn babies. Our trial aimed to use simple everyday technology to establish a program supporting a healthy lifestyle and parenting which is affordable, sustainable and potentially transferable to health services. However, the findings did not demonstrate that the women who received text messages were more likely to achieve the healthy weight, dietary and PA goals. Despite this, the intervention was reported to be engaging and satisfying, and three-quarters of the responders reported it motivated them to improve their lifestyle, with some evidence of this from the higher rates of activity monitor use in the intervention group compared to the control.

We have demonstrated that a text-messaging-based lifestyle program is effective in other patient cohorts. The TEXT ME Study delivered a similar text-messaging program to participants with coronary heart disease and demonstrated improvements in multiple clinical risk factors, including BMI and PA [10]. Some recent publications on text messaging for people with T2DM have demonstrated small improvements in HbA1c [14,15].

A major limitation affecting SMs2 was the COVID-19 pandemic. It curtailed recruitment, and COVID-19 lockdowns and restrictions reduced the opportunities for PA and access to fresh food. Only half the expected number of participants met the HLO, with dropout higher than expected. The majority of participants in the qualitative evaluation indicated that the pandemic affected their ability to maintain a healthy lifestyle. There was a period of “stay-at-home orders” that did not allow people to leave their homes except for essential reasons, and recreational facilities were closed. The Fitbit corporation recorded a global reduction in the steps taken amongst its users in the early phases of the pandemic [31]. A systematic review of population health surveys found that half of respondents gained weight during the COVID-19 pandemic: there was a 36–59% increase in food consumption and a 61–67% decrease in PA [32].

The qualitative evaluation undertaken in this study indicates that the lack of family support arising from the COVID-19 restrictions added to the burden experienced by many women, possibly further confounding our trial. A lack of family support, as well as the need to care for their baby, may have contributed to the discordance between motivation and action to improve lifestyle. Travel restrictions affected all the participants in the study and was especially difficult for the women of a migrant background who would have relied on their mothers and other family to support them after pregnancy [33]. Isolation and loss of post-partum support, including cultural practices, were the themes discerned from a qualitative survey of women post-partum during COVID-19 restrictions [34]. Our unidirectional text messages were intended to be an adjunct to, rather than a substitute for, the support from social relationships and were insufficient to induce significant positive lifestyle changes in these circumstances.

The dietary findings of this study are disappointing. Both the baseline and 6-month overall dietary intake was extremely poor, particularly in terms of vegetable intake. The failure of so few participants to consume even just three servings of vegetables a day is particularly concerning, and this was a major contributor to the lack of an effect of the intervention on the HLO. One might have expected healthier dietary habits amongst this cohort, as they had all received dietary education whilst they were pregnant. The post-partum reduction in discretionary food intake in both groups suggests that there was some positive impact. Perhaps a focus on carbohydrates obscured the messaging on vegetable intake. The baseline dietary survey indicates that this cohort had a low vegetable intake prior to pregnancy, and they potentially reverted to their usual dietary habits post-partum. This, however, is not unusual amongst Australian women with GDM. A survey conducted in the same area of Sydney by our group 15 years previously, among women 6–24 months after a GDM pregnancy, found that a mean of 2 servings of vegetables and 1.5 servings of fruit was consumed per day [30]. Another Australian study from 2012 also found that the diet quality was poor amongst women with past GDM, with participants scoring a mean of 30.9 out of a maximum of 74 on the Australian Recommended Food Score [35]. It is disappointing that the Smart Mums trial shows that fruit and vegetable intake has not improved amongst women with past GDM since that time and that the text messaging intervention did not help.

There has been recent interest in m-health dietetic interventions, but they have generally had limited effects on changing dietary habits. One online dietetic intervention for women post-GDM found that the personalisation of dietetic advice using a website, text messaging and video coaching resulted in improved self-efficacy and quality of life but there was no effect on weight, compared to access to a website with or without text messaging [36]. Another study undertaken by our group among people with macular degeneration found that a telephone-delivered dietetic intervention achieved an increase in leafy greens, but there was no difference in vegetable intake compared to the control subjects [37]. A systematic review and meta-analysis of electronic and mobile-phone-based interventions promoting vegetable and fruit intake in young adults (age 18–35) also found disappointing outcomes, with a combined improvement of only 0.2 servings a day [38].

The question arises of whether having individualised dietary counselling sessions as part of the intervention might have improved the dietary outcomes. A meta-analysis of RCTs comparing individualised dietetic consultations to the usual care found that this intervention resulted in a modest net weight reduction of 1 kg after a median of 6 months [39]. In the Smart Mums pilot study, there were two individual dietitian counselling sessions, and a reduction in carbohydrate intake was seen in the intervention group, though the overall daily energy intake was not reduced [9]. Having individual dietary counselling adds considerably to the cost of such an intervention and would limit the widespread adoption of such a program.

Our overall 38% rate of post-partum OGTTs is in line with previous Australian studies which have found follow-up rates of 27–58% [40,41] but potentially was affected by reticence to attend pathology testing and the advice from some professional organisations not to undertake a GTT during the COVID-19 pandemic and lockdowns [42,43]. In fact we felt obligated to modify our messaging to support a delay in the 6–12 week OGTT due to the COVID-19 lockdowns. There was a trend of women who received text messages undergoing a timely OGTT, but our trial was not powered to detect a difference in compliance with GTT testing.

Breastfeeding has been shown to reduce the development of T2DM after GDM [44] and improve glucose levels in subsequent pregnancies [27]. There are Australian data indicating that some 60% of babies are breastfeeding at 6 months [45]. In our study, the overall duration of breastfeeding at 6 months (84%) was even higher, perhaps limiting the ability to detect a difference with the intervention.

Unexpectedly, our survey data suggested a trend towards greater PA amongst the control subjects at 6 months. With only half the participants completing the PA survey, this is likely affected by ascertainment bias, particularly among the control group, as active women may be more likely to complete the questionnaire. The activity monitor itself may have also served as an intervention for the control group, even without feedback messages, as the survey we conducted indicated that it was a positive influence on both the intervention and control groups. This may be the reason for PA being the component of the HLO which was most likely to have been met. The activity monitor, however, provides objective data from almost the entire cohort. The higher utilisation of the activity monitor and the number of days with a higher step count amongst the intervention group are encouraging and suggest that linking text messages to an activity monitor increases their effectiveness and the sustainability of their usage. We have previously demonstrated that healthy lifestyle messages stimulate an immediate increase in activity-monitor-measured step count [46]. Other studies have demonstrated that the use of a pedometer or activity monitor facilitates an increase in daily steps and a reduction in weight after pregnancy [47,48]. Activity monitor technology is readily available, popular and a natural enhancement to texting interventions.

Apart from the challenges of conducting the trial in the midst of the COVID-19 pandemic and the potential for the activity monitor given to the control participants to act as an intervention, the major limitation of the study was the high dropout rate. Only 64% of subjects provided sufficient data to assess the primary outcome of the study. This is similar to the GEM Study, where 73% of participants did not attend for the measurement of the primary outcome of clinic-measured weight at 6 months [8]. The rate of measurement of weight has been higher in other text messaging studies, with 98% of participants having a 6-month weigh-in in a study of risk factor reduction in patients with coronary disease and 89–99% having 6-month weight data available in studies providing self-management support for people with diabetes [49,50,51]. The higher dropout rate in post-GDM studies may be related to the population, where the challenges of caring for a baby take precedence, and in SMs2, the COVID-19 pandemic added to these difficulties.

Another limitation of the study was the use of self-reported weight and basing most of the outcomes on self-reported measures. The literature is divided regarding the reliability of self-reported weight, and there are data that under-reporting is more prevalent among young women in Australia [52]. However, the positive aspect of this is that it enabled data to be collected despite the COVID-19 lockdowns. The AAQ has been well validated, but it was designed to be undertaken using computer-assisted telephone interviewing or face-to-face interviews [26]. In SMs2, the AAQ was self-completed. There are data that this may underestimate PA compared to an activity monitor [53], though the high level of PA pre-pregnancy in our study suggests that over-reporting is also a possibility.

A strength of the study is that we have included women from different cultural backgrounds and shown that the text messages were generally engaging and well received. This is important in a multicultural society such as Australia, particularly as women from migrant backgrounds are more likely to suffer from GDM [54,55] and develop diabetes in the long term [56,57]. Including culturally specific dietary information would have further strengthened the intervention, but we did not incorporate this into SMs2, as the backgrounds of the women were too diverse for a study of this scale to cater to. The incorporation of messages about breastfeeding is a novel aspect of a post-partum intervention to reduce diabetes. The importance of breastfeeding to maternal metabolic health and reductions in diabetes risk has been under-appreciated [44].

The lessons learned from the current trial will facilitate the refinement of text messaging interventions for women after GDM. It may be that women who are more likely to be engaged by text messaging and those who are at the action, contemplation or preparation stages of readiness according to the Transtheoretical Model may be most suitable [58]. Other factors related to parenthood, such as parity; support from partners, family and friends or just being too busy are factors which potentially affect PA participation and self-efficacy [59,60,61,62]. Having additional health professional support in the program would likely be helpful but adds to the cost and reduces the generalisability of programs. However, even with face-to-face lifestyle interventions, RCTs have had mixed results in reducing diabetes or its risk factors [7,8]. One other consideration is whether a lifestyle intervention program should be delayed until some years later, when the mother can devote more time to her own lifestyle habits. However, meta-analyses currently suggest that interventions commenced soon after delivery are more effective [6,63].

With confounding due to the COVID-19 pandemic at multiple levels, it is not possible to draw conclusions about the effectiveness of a text messaging program linked to activity monitors to support a healthy lifestyle after GDM. However, our study demonstrates a proof of concept that participants were engaged and found it motivating and that tailoring of PA messages through the linkage of activity monitors is feasible, with increases in activity monitor utilisation achieved. How best to deliver the dietary component of the intervention remains unclear as this must be feasible and affordable if we are to have a widely implemented program. Further studies in a more favourable environment need to be undertaken to determine whether such a program can reduce diabetes risk.

## Figures and Tables

**Table 1:** Participant characteristics: baseline and pregnancy.

	Intervention (n = 88)	Control (n = 89)	Overall (n = 177)
Age (years)	32.3 ± 4.5	32.2 ± 4.8	32.2 ± 4.6
Pre-pregnancy Body Mass Index (kg/m^2^)	29.3 ± 7.4	28.3 ± 6.4	28.8 ± 6.9
Gestational age at booking in (weeks)	17.5 ± 4.9	17.2 ± 4.9	17.3 ± 4.9
Ethnicity			
Australian/New Zealand (non-Indigenous)	21 (24%)	19 (21%)	40 (23%)
South Asian	40 (46%)	43 (48%)	83 (47%)
East and Southeast Asian	4 (5%)	12 (13%)	16 (9%)
Others	23 (26%)	15 (17%)	38 (21%)
Marital status			
Married/de facto	80 (90.9%)	84 (94.4%)	164 (93%)
Single/separated/widowed	8 (9%)	5 (6%)	13 (7%)
Education			
Tertiary	62 (70%)	73 (82%)	135 (76%)
Secondary or less	26 (30%)	16 (19%)	42 (24%)
Employment status before pregnancy			
Working full time	44 (50%)	44 (49%)	88 (50%)
Working part time	24 (27%)	28 (32%)	52 (29%)
Not in paid work	20 (22%)	17 (19%)	37 (21%)
Background medical history			
Polycystic ovary syndrome	16 (18%)	10 (11%)	26 (15%)
Depression	8 (9%)	9 (10%)	17 (10%)
Hypertension	3 (3%)	6 (7%)	9 (5%)
Hypercholesterolaemia	5 (6%)	5 (6%)	10 (6%)
Previous gestational diabetes	28 (32%)	29 (33%)	57 (32%)
Family history of diabetes (first degree)	47 (53%)	66 (63%)	103 (58%)
Primiparous	29 (33%)	37 (42%)	66 (37%)
Multiparous	59 (67%)	52 (58%)	111 (63%)
Breastfeeding of previous children	54/59 (92%)	48/52 (92%)	102/111 (92%)
Smoking			
Current smoker	2 (2%)	4 (5%)	6 (3%)
Former smoker	13 (15%)	13 (15%)	26 (15%)
Never smoked	73 (83%)	72 (81%)	145 (82%)
Drinks alcohol when not pregnant	29 (33%)	13 (15%)	42 (24%)
Vegetarian	14 (16%)	16 (18%)	30 (17%)
Total PA time prepregnancy (min/week)	340 ± 334	336 ± 344	338 ± 338
Diet prepregnancy			
Servings of vegetables per day	1.2 ± 0.9	1.1 ± 0.9	1.1 ± 0.9
Servings of fruit per day	1.1 ± 0.7	1.0 ± 0.7	1.1 ± 0.7
Servings of discretionary food per week	19.3 ± 12.4	16 ± 10.5	17.6 ± 11.6
Alcoholic drinks per day	0.1 ± 0.2	0.0 ± 0.1	0.1 ± 0.2
Weeks gestation at delivery	37.5 ± 2.3	37.1 ± 3.1	37.3 ± 2.8
Birth weight (g)	3381 ± 548	3236 ± 509	3308 ± 532
Mode of delivery			
Caesarean section	42 (48%)	33 (37%)	75 (42%)
Instrument	7 (8%)	12 (14%)	19 (11%)
Normal vaginal delivery	39 (44%)	44 (49%)	83 (47%)
GDM treatment during pregnancy			
Insulin	46 (52%)	47 (53%)	93 (53%)
Metformin	12 (14%)	6 (7%)	18 (10%)
Maternal complications			
Pre-eclampsia	3 (3%)	5 (6%)	8 (5%)
Post-partum haemorrhage	11 (13%)	6 (7%)	17 (10%)
3rd- or 4th-degree vaginal tear	1 (1%)	2 (2%)	3 (2%)
Admission to neonatal intensive care	4 (5%)	9 (10%)	13 (7%)

**Table 2:** Outcomes at 6 months (adjusted to baseline).

	Intervention	Control	Adjusted Relative Risk (95%CI) or Mean Difference (95%CI)	*p* Value
Primary Outcome				
Met Healthy Lifestyle Outcome *	7/57 (12%)	6/56 (11%)	1.15 (0.41–3.2)	0.79
Secondary Outcomes				
Met weight goal	20/67 (30%)	12/62 (19%)	1.54 (0.82–2.89)	0.17
Met physical activity goal	23/49 (47%)	29/46 (63%)	0.72 (0.50–1.04)	0.08
Met dietary goal	2/58 (3%)	0/58 (0%)	N/A	N/A
Body Mass Index (kg/m^2^)	29.4 ± 6.9	28.4 ± 5.9	0.97 (−1.24–3.18)	0.39
Total physical activity time (mins/week)	260 ± 278 (N = 49)	301 ± 262 (N = 46)	−41 (−150–68)	0.46
OGTT performed by 12 weeks post-partum	38/5 (43%)	29/89 (33%)	1.22 (0.78–1.91)	0.36
Breastfeeding at 1 month Yes Intensity (BLISS Score)	46/50 (92%) 5.1 ± 1.8	48/54 (89%) 4.5 ± 2.2	1.04 (0.91–1.17) 0.66 (−0.12–1.44)	0.60 0.20
Any breastfeeding at 6 months Yes Intensity (BLISS Score)	34/44 (77%) 20.4 ± 4.3	41/45 (91%) 19.3 ± 5.5	0.85 (0.71–1.02) 1.06 (−1.00–3.13)	0.07 0.52
EPDS score ≥ 10	15/88 (17%)	12/89 (14%)	1.26 (0.63–2.54)	0.51

* Healthy Lifestyle Outcome was met if at least two of the following three components were achieved: 1. Weight: Reaching pregravid weight if pregravid BMI was <25 kg/m^2^ or losing ≥ 5% of pregravid weight if pregravid BMI was ≥25 kg/m^2^; 2. PA: Whether the Australian guidelines of 150 min of moderate-intensity PA each week had been met; 3. Diet: Whether 1 serving of fruit and 3 servings of vegetables were consumed per day and discretionary foods were consumed ≤14 times a week. OGTT = oral glucose tolerance test, BLISS = Breastfeeding Length Intensity Scoring System, EPDS = Edinburgh Postnatal Depression Score.

**Table 3:** Dietary outcomes at 6 months.

	Intervention n = 58	Control n = 58	Adjusted Relative Risk (95%CI) or Mean Difference (95%CI)	*p* Value
Met Healthy Lifestyle Outcome dietary goal	2 (3%)	0 (0%)	N/A	N/A
Met Australian Dietary Guidelines dietary goal	0 (0%)	0 (0%)	N/A	N/A
Servings of vegetables per day	1.2 ± 0.8 *	1.0 ± 0.6	1.04 (−0.87–2.95)	0.29
Met vegetable goal	4 (7%) *	0 (0%)	N/A	N/A
Servings of fruit per day	0.7 ± 0.4	0.7 ± 0.4	0.00 (−1.23–1.23)	1.00
Met fruit goal	21 (36%)	21 (36%)	0.93 (0.58–1.46)	0.74
Servings of discretionary foods per week	13.6 ± 8.2 **	11.4 ± 6.1	2.19 (−0.45–4.84)	0.11
Met discretionary foods goal	33 (59%) **	40 (69%)	1.02 (0.84–1.23)	0.85
Servings of alcohol per day	0.1 ± 0.2	0.0 ± 0.1	0.22 (−0.14–0.59)	0.24

* Data complete for 57 subjects; ** data complete for 56 subjects.

## Data Availability

The data presented in this study are available on request from the corresponding author. The data are not publicly available due to [privacy concerns].

## Supplementary Materials

The following supporting information can be downloaded at: https://www.mdpi.com/article/10.3390/nu16060820/s1, Table S1. Participants by completeness of primary outcome (i.e., having weight, physical activity and diet data for assessment of Healthy Lifestyle Outcome). Table S2. Example comments related to enablers and barriers.

## References

[1] Sweeting A., Wong J., Murphy H.R., Ross G.P. (2022). A clinical update on gestational diabetes mellitus. Endocr. Rev..

[2] Kim C., Newton K.M., Knopp R.H. (2002). Gestational diabetes and the incidence of type 2 diabetes. Diabetes Care.

[3] Vounzoulaki E., Khunti K., Abner S.C., Tan B.K., Davies M.J., Gillies C.L. (2020). Progression to type 2 diabetes in women with a known history of gestational diabetes: Systematic review and meta-analysis. BMJ.

[4] Cheung N.W., Byth K. (2003). The population health significance of gestational diabetes. Diabetes Care.

[5] Ratner R.E., Christophi C.A., Metzger B.E., Dabelea D., Bennett P.H., Pi-Sunyer X., Fowler S., Kahn S.E., The Diabetes Prevention Program Research Group (2008). Prevention of diabetes in women with a history of gestational diabetes: Effects of metformin and lifestyle interventions. J. Clin. Endocrinol. Metab..

[6] Li N., Yang Y., Cui D., Li C., Ma R.C., Li J., Yang X. (2021). Effects of lifestyle intervention on long-term risk of diabetes in women with prior gestational diabetes: A systematic review and meta-analysis of randomized controlled trials. Obes. Rev..

[7] Tandon N., Gupta Y., Kapoor D., Lakshmi J.K., Praveen D., Bhattacharya A., Billot L., Naheed A., de Silva A., Gupta I. (2022). Effects of a lifestyle intervention to prevent deterioration in glycemic status among South Asian women with recent gestational diabetes: A randomized clinical trial. JAMA Netw. Open.

[8] Ferrara A., Hedderson M.M., Brown S.D., Albright C.L., Ehrlich S.F., Tsai A.L., Caan B.J., Sternfeld B., Gordon N.P., Schmittdiel J.A. (2016). The comparative effectiveness of diabetes prevention strategies to reduce postpartum weight retention in women with gestational diabetes mellitus: The Gestational Diabetes’ Effects on Moms (GEM) cluster randomized trial. Diabetes Care.

[9] Cheung N.W., Blumenthal C., Smith B.J., Hogan R., Thiagalingam A., Redfern J., Barry T., Cinnadaio N., Chow C.K. (2019). A pilot randomised controlled trial of a text messaging intervention with customisation using linked data from wireless wearable activity monitors, to improve risk factors following gestational diabetes. Nutrients.

[10] Chow C.K., Redfern J., Hills G.S., Thakkar J., Santo K., Hackett M.L., Jan S., Graves N., de Keizer L., Barry T. (2015). Effect of lifestyle-focused text messaging on risk factor modification in patients with coronary heart disease: A randomized clinical trial. JAMA.

[11] Skinner R., Gonet V., Currie S., Hoddinot P., Dornbrowski S.U. (2020). A systematic review with meta-analyses of text message-delivered behaviour change interventions for weight loss and weight loss maintenance. Obes. Rev..

[12] Smith D.M., Duque L., Huffman J.C., Healy B.C., Celano C.M. (2019). Text message interventions for physical activity: A systematic review and meta-analysis. Am. J. Prevent Med..

[13] Scott-Sheldon L.A., Lantini R., Jennings E.G., Thind H., Rosen R.K., Salmoirago-Blotcher E., Bock B.C. (2016). Text messaging-based interventions for smoking cessation: A systematic review and meta-analysis. JMIR Mhealth Uhealth.

[14] Dobson R., Whittaker R., Jiang Y., Maddison R., Shepherd M., McNamara C., Cutfield R., Khanolkar M., Murphy R. (2018). Effectiveness of text message based, diabetes self management support programme (SMS4BG): Two arm, parallel randomised controlled trial. Br. Med. J..

[15] Nelson L.A., Greevy R.A., Spieker A., Wallston K.A., Elasy T.A., Kripalani S., Gentry C., Bergner E.M., LeStourgeon L.M., Williamson S.E. (2021). Effects of a tailored text messaging intervention among diverse adults with type 2 diabetes: Evidence from the 15-month REACH randomized controlled trial. Diabetes Care.

[16] Burn E., Nghiem S., Jan S. (2017). Cost-effectiveness of a text-message program for cardiovascular disease secondary prevention. Heart.

[17] Marschner S., Chow C., Thiagalingam A., Simmons D., McLean M., Pasupathy D., Smith B.J., Flood V., Padmanabhan S., Melov S. (2021). Effectiveness of a customised mobile phone text messaging intervention supported by data from activity monitors for improving lifestyle factors related to the risk of type 2 diabetes among women after gestational diabetes: Protocol for a multicentre randomised controlled trial (SMART MUMS with smart phones 2). BMJ Open.

[18] World Health Organization Consultation (2014). Diagnostic criteria and classification of hyperglycaemia first detected in pregnancy: A World Health Organization Guideline. Diabetes Res. Clin. Pract..

[19] Martin F.I.R. (1991). The diagnosis of gestational diabetes. Med. J. Aust..

[20] Carey R.N., Connell L.E., Johnston M., Carey R.N., Connell L.E., Johnston M., Rothman A.J., de Bruin M., Kelly M.P., Michie S. (2019). Behavior change techniques and their mechanisms of action: A synthesis of links described in published intervention literature. Ann. Behav. Med..

[21] NHMRC (2013). Australian Dietary Guidelines.

[22] Szeto M.D., Barber C., Ranpariya V.K., Anderson J., Hatch J., Ward J., Aguilera M.N., Hassan S., Hamp A., Coolman T. (2022). Emojis and emotions in health care and dermatology communication: Narrative review. JMIR Dermatol..

[23] Nankervis A., McIntyre H.D., Moses R., Ross G.P., Callaway L., Porter C., Jeffries W., Boorman C., De Vries B., McElduff, A. for the Australasian Diabetes in Pregnancy Society ADIPS Consensus Guidelines for the Testing and Diagnosis of Hyperglycaemia in Pregnancy in Australia and New Zealand. http://www.adips.org/downloads/adipsconsensusguidelinesgdm-03.05.13versionacceptedfinal.pdf.

[24] Adams M.A., Sallis J.F., Normal G.J., Hovell M.F., Hekler E.B., Perata E. (2013). An adaptive physical activity intervention for overweight adults: A randomized controlled trial. PLoS ONE.

[25] Galbicka G. (1994). Shaping in the 21st century: Moving percentile schedules into applied settings. J. App. Behav. Anal..

[26] Australian Institute of Health and Welfare (AIHW) (2003). The Active Australia Survey: A Guide and Manual for Implementation, Analysis and Reporting.

[27] Melov S.J., White S.J., Simmons M., Kirby A., Stulz V., Padmanabhan S., Alahakoon T.I., Pasupathy D., Cheung N.W. (2022). The BLIiNG Study—Breastfeeding length and intensity in gestational diabetes and metabolic effects in a subsequent pregnancy: A cohort study. Midwifery.

[28] Cox J.L., Holden J.M., Sagovsky R. (1987). Detection of postnatal depression. Development of the 10-item Edinburgh Postnatal Depression Scale. Br. J. Psychiatry.

[29] Smith B.J., Cheung N.W., Zehle K., McLean M., Bauman A.E. (2005). Post-partum physical activity and related psychosocial factors among women with recent gestational diabetes. Diabetes Care.

[30] Zehle K., Smith B.J., Chey T., McLean M., Bauman A.E., Cheung N.W. (2008). Psychosocial factors related to diet among women with recent gestational diabetes: Opportunities for intervention. Diab. Educ..

[31] Business World. https://www.bworldonline.com/community/2020/04/02/287449/sparkup-community-fitbit-data-reveals-the-impact-of-covid-19-on-global-activity/.

[32] Chew H.S.J., Lopez V. (2021). Global Impact of COVID-19 on weight and weight-related behaviors in the adult population: A scoping review. Int. J. Environ. Res. Public Health.

[33] Melov S., Galas N., Swain J., Alahakoon T.I., Lee V., Cheung N.W., McGee T., Pasupathy D., McNab J. (2023). Women’s experience of perinatal support in a high migrant Australian population during the COVID-19 pandemic: A mixed methods study. BMC Preg. Childbirth.

[34] Bayrampour H., Tsui M.Y.E. (2023). Postpartum people’s experiences of and responses to the COVID-19 pandemic during the first year of the pandemic: A descriptive qualitative study. Women’s Health.

[35] Morrison M.K., Koh D., Lowe J.M., Miller Y.D., Marshall A.L., Colyvas K., Collins C.E. (2012). Postpartum diet quality in Australian women following a gestational diabetes pregnancy. Eur. J. Clin. Nutr..

[36] Taylor R., Rollo M.E., Baldwin J.N., Hutchesson M., Aguiar E.J., Wynne K., Young A., Callister R., Collins C.E. (2022). Evaluation of a type 2 diabetes risk reduction online program for women with recent gestational diabetes: A randomised trial. Int. J. Behav. Nutr. Phys. Act..

[37] Tang D., Mitchell P., Liew G., Burlutsky G., Flood V.M., Gopinath B. (2020). Telephone-delivered dietary intervention in patients with age-related macular degeneration: 3-month post-intervention findings of a randomised controlled trial. Nutrients.

[38] Nour M., Chen J., Allman-Farinelli M. (2016). Efficacy and external validity of electronic and mobile phone-based interventions promoting vegetable intake in young adults: Systematic review and meta-analysis. J. Med. Internet Res..

[39] Williams L.T., Barnes K., Ball L., Ross L.J., Sladdin I., Mitchell L.J. (2019). How effective are dietitians in weight management? A systematic review and meta-analysis of randomized controlled trials. Healthcare.

[40] Morrison M.K., Collins C.E., Lowe J.M. (2009). Postnatal testing for diabetes in Australian women following gestational diabetes mellitus. Aust. N. Z. J. Obstet. Gynaecol..

[41] Boyle D.I.R., Versace V.L., Dunbar J.A., Scheil W., Janus E., Oats J.J.N., Skinner T., Shih S., O’Reilly S., Sikaris K. (2018). Results of the first recorded evaluation of a national gestational diabetes mellitus register: Challenges in screening, registration, and follow-up for diabetes risk. PLoS ONE.

[42] Australasian Diabetes in Pregnancy Society (ADIPS), the Australian Diabetes Society (ADS), the Australian Diabetes Educators Association (ADEA), Diabetes Australia (DA) Diagnostic Testing for Gestational Diabetes Mellitus (GDM) during the COVID 19 Pandemic: Antenatal and Postnatal Testing Advice. https://www.adips.org/documents/RevisedGDMCOVID-19GuidelineFINAL30April2020pdf_000.pdf.

[43] Yamamoto J., Donovan L., Feig D., Berger H. Temporary Alternative Screening Strategy for Gestational Diabetes Screening during the COVID-19 Pandemic. A Joint Consensus Statement from the Diabetes Canada Clinical Practice Guidelines Steering Committee and the Society of Obstetricians and Gynecologists of Canada. https://els-jbs-prod-cdn.jbs.elsevierhealth.com/pb/assets/raw/health%20advance/journals/jcjd/jcjd_covid_guidelines_020420.pdf.

[44] Gunderson E.P., Hurston S.R., Ning X., Lo J.C., Crites Y., Walton D., Dewey K.G., Azevedo R.A., Young S., Fox G. (2015). Lactation and progression to type 2 diabetes mellitus after gestational diabetes mellitus: A prospective cohort study. Ann. Intern. Med..

[45] Netting M.J., Moumin N.A., Knight E.J., Golley R.K., Makrides M., Green T.J. (2022). The Australian feeding infants and toddler study (OzFITS 2021): Breastfeeding and early feeding practices. Nutrients.

[46] Cheung N.W., Thiagalingam A., Smith B.J., Redfern J., Barry T., Mercorelli L., Chow C.K. (2022). Text messages promoting healthy lifestyle and linked with activity monitors stimulate an immediate increase in physical activity among women after gestational diabetes. Diabetes Res. Clin. Pract..

[47] Peacock A.S., Bogossian F.E., Wilkinson S.A., Gibbons K.S., Kim C., McInthyre H.D. (2015). A randomised controlled trial to delay or prevent type 2 diabetes after gestational diabetes: Walking for exercise and nutrition to prevent diabetes for you. Int. J. Endocrinol..

[48] Maturi M.S., Afshary P., Abedi P. (2011). Effect of physical activity intervention based on a pedometer on physical activity level and anthropometric measures after childbirth: A randomized controlled trial. BMC Pregnancy Childbirth.

[49] Huo X., Krumholz H.M., Bai X., Spatz E.S., Ding Q., Horak P., Zhao W., Gong Q., Zhang H., Yan X. (2019). Effects of mobile text messaging on glycemic control in patients with coronary heart disease and diabetes mellitus. Circ. Cardiovasc. Qual. Outcomes.

[50] Waller K., Furber S., Bauman A., Allman-Farinelli M., van den Dolder P., Hayes A., Facci F., Franco L., Webb A., Moses R. (2021). Effectiveness and acceptability of a text message intervention (DTEXT) on HbA1c and self-management for people with type 2 diabetes. A randomized controlled trial. Patient Educ. Couns..

[51] Cheung N.W., Redfern J., Thiagalingam A., Hng T.M., Marschner S., Haider R., Faruquie S., von Huben A., She S., McIntyre D. (2023). Effect of mobile phone text messaging self-management support for patients with diabetes or coronary heart disease in a chronic disease management program (SupportMe) on blood pressure: Pragmatic randomized controlled trial. J. Med. Internet Res..

[52] Bowring A.L., Peeters A., Freak-Poli R., Lim M.S.C., Gouillou M., Hellard M. (2012). Measuring the accuracy of self-reported height and weight in a community-based sample of young people. BMC Med. Res. Methodol..

[53] Quinlan C., Rattray B., Pryor D., Northey J.M., Antsey K.J., Butterworth P., Cherbuin N. (2021). The accuracy of self-reported physical activity questionnaires varies with sex and body mass index. PLoS ONE.

[54] Head S.H., Rosella L.C., Berger H., Feig D.S., Fleming K., Ray J.G., Shah B.R., Lipscombe L.L. (2021). BMI and risk of gestational diabetes among women of south Asian and Chinese ethnicity: A population-based study. Diabetologia.

[55] Wong V.W., Lin A., Russell H. (2017). Adopting the new World Health Organization diagnostic criteria for gestational diabetes: How the prevalence changes in a high-risk region in Australia. Diabetes Res. Clin. Pract..

[56] Lee A.J., Hiscock R.J., Wein P., Walker S.P., Permezel M. (2007). Gestational diabetes mellitus: Clinical predictors and long-term risk of developing type 2 diabetes. Diabetes Care.

[57] Oldfield M.D., Donley P., Walwyn L., Scudamore I., Gregory R. (2007). Long term prognosis of women with gestational diabetes in a multiethnic population. Postgrad. Med. J..

[58] Prochaska J.O. (1984). Systems of Psychotherapy: A Transtheoretical Analysis.

[59] Hill B., McPhie S., Skouteris H. (2016). The role of parity in gestational weight gain and postpartum weight retention. Womens Health Issues.

[60] Blum J.W., Beaudoin C.M., Caton-Lemos L. (2004). Physical activity patterns and maternal well-being in post-partum women. Matern. Child Health J.

[61] Salingeh M., McNamara B., Rooney R. (2016). Perceived barriers and enablers of physical activity in postpartum women: A qualitative approach. BMC Pregnancy Childbirth.

[62] Albright C., Saiki K., Steffen A.D., Wofkel E. (2015). What barriers thwart postpartum women’s physical activity goals during a 12-month intervention? A process evaluation of the Na Mikimiki project. Women Health.

[63] Goveia P., Canon-Montanez W., de Paula Santos D., Lopes G.W., Ma R.C. (2018). Lifestyle intervention for the prevention of diabetes in women with previous gestational diabetes mellitus. A systematic review and meta-analysis. Front. Endocrinol..

