# Supplementary material for: Randomised Controlled Trial of a Customised Text Messaging and Activity Monitor Program for Lifestyle Improvement after Gestational Diabetes
Source: Nutrients. 2024 Mar 13;16(6):820. doi: 10.3390/nu16060820 (PMC10974605; doi:10.3390/nu16060820)
Supplement: Supplementary file 1 [file nutrients-16-00820-s001.zip › nutrients-2822815-supplementary.pdf]

nutrients

MDPI

Table S1. Participants by completeness of primary outcome (ie having weight, physical activity and diet data for assessment of Healthy Lifestyle Outcome).

	Complete N=113	Incomplete n=64	*p* value
Age (years)	33.1±4.1	30.7±5.1	<0.01
Pre-pregnancy Body Mass Index (kg/m^2)	27.6±6.1	31.0±7.8	<0.01
Ethnicity			<0.01
Australian/New Zealand (non-Indigenous)	15 (13.3%)	25 (39.1%)	
Other	97 (85.8%)	39 (60.9%)	
Marital status			0.23
Married/Defacto	107 (94.7%)	57 (89.1%)	
Single/Separated/Widowed	6 (5.3%)	7 (10.9%)	
Education			<0.01
Secondary or less	16 (14.2%)	26 (40.6%)	
Tertiary (TAFE/Uni)	97 (85.8%)	38 (59.4%)	
Employment			0.38
Work full time	59 (52.2%)	29 (45.3%)	
Work part time	34 (30.1%)	18 (28.1%)	
Not in paid work	20 (17.7%)	17 (26.6%)	
History of gestational diabetes	1.3±0.6	1.3±0.6	0.79
Family history of diabetes (first degree)	68/113 (60.2%)	35/64 (54.7%)	0.53
Gravidity	2.6±1.7	2.8±2.1	0.56
Parity	0.9±0.8	1.1±1.2	0.45
Smoking			<0.01
Never smoked	103/113 (91.2%)	42/64 (65.6%)	
Former smokers	7/113 (6.2%)	19/64 (29.7%)	
Current smokers	3/113 (2.7%)	3/64 (4.7%)	
Drinks alcohol when not pregnant	29/113 (25.7%)	13/64 (20.3%)	0.47
Total PA time prepregnancy (min/week)	336.9±303.7	339.9±401.0	0.36
Gestational weeks at delivery	37.3±3.3	37.4±1.6	0.13
Birth Weight (g)	3306.1±518.4	3311.5±559.5	0.74
Mode of delivery			0.95
Caesarean section	49/113 (43.4%)	26 / 64 (40.6%)	
Instrument	12/113 (10.6%)	7/64 (10.9%)	
Normal vaginal	52/113 (46.0%)	31/64 (48.4%)	
Insulin treatment during pregnancy	60 / 113 (53.1%)	33 / 64 (51.6%)	0.88
Metformin during pregnancy	12/113 (10.6%)	6/64 (9.4%)	1.0

Table S2. Example comments related to enablers and barriers.

**Examples of Enabler Comments**
"It just kept reinforcing stay on track, remember you're still at risk [for diabetes]. So it just kept me conscious of that and I actually found that really, really helpful" (Qualitative Participant 7)
"…definitely for me it is very helpful, so I will recommend for others also" (Qualitative Participant 11)
"Like every week I had a target to reach. Sometimes I was going above that and I will get really motivated to reach my target. And instead of just sort of sitting and watching TV, I was trying to go out with my kids." (Qualitative Participant 10)
"… it's kind of giving me some sort of discipline. And then the regular messages, I basically read and then it's top on my mind. So what should I eat and what should I not? Yeah, …. It was beneficial" (Qualitative Participant 13)
"I'm a bit sad that it's over. Yeah, I think it's great experience. I find it really valuable, and I do hope that it does continue because just creating that awareness and that consciousness, I think it's really, really important" (Qualitative Participant 7)
"Like they ask how vigorous exercise we was (sic) doing and then once I saw the survey, I was expected to do maybe it's safe to do it, then I started after survey to do more like walking and even running a little bit" (Qualitative Participant 10)
"[the surveys] would just highlight the fact that I need to make better choices no matter what I had around me. I can still make a healthy choice even though we have an x and y in the house. I can still make a healthy choice no matter what" (Qualitative Participant 6)
**Examples of Barrier Comments**
"except for my husband, there was no one [for support]" (Qualitative Participant 12)
"I'm still trying to find that space or that time for me because I don't get any alone time except grocery shopping" (Qualitative Participant 7)
"So mostly I do nightshifts, so it was really hard for me to get up in the morning, do some exercises, and eat my veggies and the proteins. … it was good reminder with the text message. It's really good" (Qualitative Participant 3)

"I am too busy, too busy. My husband going work and then I'm alone at home with a baby and then there's so many things. I just rather don't care my health feel like that because it's too busy, it's too hectic" (Qualitative Participant 13)

"I guess I kind of put myself last with everything with my family." (Qualitative Participant 8)

"I had a caesarean … for quite a few months, like even into this year, I still had some pain from the stitches" (Qualitative Participant 4)

"Well for me I tore pretty bad... So for me, exercise straight out was not a thing… like maybe two and a half, three [months]… I probably wouldn't have done anything prior…I also have a hernia from giving birth to her as well. So I am not comfortable" (Qualitative Participant 8)

Related to COVID-19

"I'm scared because my son is small and I'm also vulnerable during that time because I'm very weak. I mean after delivery, physically, yeah, we don't have that much energy and that like immunity also, right?... Like for my child and for my safety, didn't go out." (Qualitative Participant 13)

"…the exercise, well that was sort of hit and miss with COVID and home schooling. And I think that sort of made it harder than anything else…It was a struggle. When she was born I had a year one and a year 8." (Qualitative Participant 4)
